# Fabrication of Sodium Trimetaphosphate-Based PEDOT:PSS Conductive Hydrogels

**DOI:** 10.3390/gels10020115

**Published:** 2024-02-01

**Authors:** Madelyn Reynolds, Lindsay M. Stoy, Jindi Sun, Prince Emmanuel Opoku Amponsah, Lin Li, Misael Soto, Shang Song

**Affiliations:** 1 Department of Biomedical Engineering, College of Engineering, University of Arizona, Tucson, AZ 85719, USA; madelynreynolds@arizona.edu (M.R.); lmstoy@arizona.edu (L.M.S.); jindisun@arizona.edu (J.S.); princeo@arizona.edu (P.E.O.A.); linl1@arizona.edu (L.L.); m.soto@colostate.edu (M.S.); 2 Departments of Materials Science and Engineering, Neuroscience GIDP, and BIO5 Institute, University of Arizona, Tucson, AZ 85719, USA

**Keywords:** conductive hydrogel, polythiophene-poly-(3,4-ethylenedioxythiophene) and polystyrene sulfonate (PEDOT:PSS), glycerol, sodium trimetaphosphate (STMP), crosslinking, conductivity, rheology

## Abstract

Conductive hydrogels are highly attractive for biomedical applications due to their ability to mimic the electrophysiological environment of biological tissues. Although conducting polymer polythiophene-poly-(3,4-ethylenedioxythiophene) (PEDOT) and polystyrene sulfonate (PSS) alone exhibit high conductivity, the addition of other chemical compositions could further improve the electrical and mechanical properties of PEDOT:PSS, providing a more promising interface with biological tissues. Here we study the effects of incorporating crosslinking additives, such as glycerol and sodium trimetaphosphate (STMP), in developing interpenetrating PEDOT:PSS-based conductive hydrogels. The addition of glycerol at a low concentration maintained the PEDOT:PSS conductivity with enhanced wettability but decreased the mechanical stiffness. Increasing the concentration of STMP allowed sufficient physical crosslinking with PEDOT:PSS, resulting in improved hydrogel conductivity, wettability, and rheological properties without glycerol. The STMP-based PEDOT:PSS conductive hydrogels also exhibited shear-thinning behaviors, which are potentially favorable for extrusion-based 3D bioprinting applications. We demonstrate an interpenetrating conducting polymer hydrogel with tunable electrical and mechanical properties for cellular interactions and future tissue engineering applications.

## 1. Introduction

Conductive polymers are known for their electrical properties and biocompatibility, enabling them to serve as neural-interfacing materials [1,2,3,4,5]. They share common chemical structures in which a series of alternating single and double bonds with overlapping pi-bonds enables the free movement of electrons between atoms [6]. The addition of dopants can significantly change the material properties of conductive polymers during the polymerization process. As an example, sodium dodecylbenzenesulfonate (NaDBS) alters the crosslinking conditions and increases material conductivity [6,7,8], resulting in polypyrrole (PPy) with drastically different mechanical properties [9,10,11,12,13] (e.g., enhanced pyrrole water solubility and roughness). Conductive hydrogels consist of three-dimensional (3D) hydrophilic networks of conductive polymers and other biocompatible materials that exhibit high strength, elasticity, and self-healing [14,15,16]. Conductive hydrogels offer more advantages than conventional polymers because they exhibit higher water content with better elastic properties while allowing sufficient conductivity [17,18]. Conductive hydrogels have been shown to be excellent candidates for tissue engineering applications, as they provide 3D structures that mimic soft tissues and exhibit desirable biocompatibility [19,20]. Stem cells cultured with conductive scaffolds experience electrical stimulation without hindered signal transduction and changes in viability [3,21]. Furthermore, conductive hydrogel-based composites are capable of adapting to physiological tissue strain and growth at nerve interfaces due to their elasticity and self-healing behavior [22,23].

Polythiophene-poly-(3,4-ethylenedioxythiophene) (PEDOT) joined with polyelectrolyte polystyrene sulfonate (PSS) is a well-known conductive and biocompatible material that is widely used for neural interfacing [16,22,23,24,25,26]. PEDOT:PSS produces a polymerization complex in water, which is favorable for biomedical fabrication. Furthermore, higher concentrations of PEDOT:PSS are correlated with increased electrical conductivity [25,26,27]. The material properties of PEDOT:PSS can be further adjusted using additives, such as glycerol, to alter its mechanical strength, durability, and conductivity [26,27,28,29]. Crosslinking with other highly biocompatible hydrogels can create interpenetrating conductive hydrogels, which mimic the elastic modulus of natural tissue [22,30,31,32] and exhibit cytocompatibility [33,34]. The addition of PSS in conductive hydrogels demonstrated higher flexibility compared to those with hyaluronic acid [6]. Interpenetrating conductive hydrogels made of gelatin methacryloyl and PEDOT:PSS induced the expression of brain-derived neurotrophic factor and neurotrophin-3 in dorsal root ganglion cells [35]. Biohybrid conductive hydrogels composed of collagen, alginate, and PEDOT:PSS enhanced sarcomere organization and beating in human induced pluripotent stem cell-derived cardiomyocytes [36]. The carboxymethyl chitosan–gelatin–PEDOT:PSS hydrogels demonstrated a compressive modulus comparable to native rat brain tissue [37]. The highly conductive and biocompatible nature of PEDOT:PSS is attractive for tissue engineering applications due to its versatility in forming interpenetrating polymer networks with other biomaterials [38,39].

Here, we investigated the effects of combining a small-molecule additive called glycerol and an inorganic crosslinker called sodium trimetaphosphate (STMP) to optimize PEDOT:PSS-based conductive hydrogels. Glycerol is known to increase the temperature tolerance, fluid retention, and elasticity of hydrogels [40,41,42]. Our previous study showed that glycerol increased the conductivity and flexibility of PEDOT:PSS-based soft electronics [22]. Furthermore, glycerol forms strong hydrogen bonds, thereby increasing the ability of the hydrogels to retain fluid [40]. STMP is a cyclic triphosphate synthesized by pyrophosphate and phosphoric acid at high temperature [43]. It has been used to crosslink biodegradable starch films for enhanced tensile strength, opacity, and solubility due to the formation of phosphodiester bonds [44,45]. Importantly, it is a non-toxic crosslinking agent suitable for physical polymers and composite biopolymer films [44,45]. STMP is also known to improve the strength and elastic modulus of composite hydrogels [46,47,48,49] and can serve as an ionic binder [50,51]. We consider that an ideal conductive hydrogel for biomedical applications needs to take advantage of dopants and crosslinking polymers to retain its electrical properties while tuning its tensile strength and elastic modulus [52,53,54,55,56,57]. We investigated whether STMP and glycerol could be individually adjusted in PEDOT:PSS-based hydrogels to achieve enhanced electrical and mechanical properties. Our results demonstrated that glycerol had no significant impact on PEDOT:PSS conductivity at low concentration, whereas STMP improved the mechanical properties of PEDOT:PSS at high concentration. Although both significantly enhanced hydrogel wettability, STMP led to a higher storage modulus and exhibited shear-thinning viscosities, which are potentially favorable for extrusion-based 3D bioprinting applications. In summary, our results unveil an interpenetrating conducting polymer hydrogel with tunable electrical and mechanical properties. The STMP-based PEDOT:PSS conductive hydrogels can serve as a new biomaterial, offering mechanical and electrical cues for interactions with cells in a variety of future tissue engineering applications.

## 2. Results and Discussion

We fabricated the interpenetrating PEDOT:PSS conductive hydrogels with glycerol and STMP at various concentrations (Table 1) using physical crosslinking mechanisms. A total of five formulations were investigated, including PEDOT:PSS alone (NPN), PEDOT:PSS with added glycerol (LPN), PEDOT:PSS with added STMP at low and high concentrations (NPL and NPH), and PEDOT:PSS with combined glycerol and STMP (LPH), for their morphology, conductivity, wettability, and rheological properties (Table 1). Glycerol was not investigated at high concentrations in PEDOT:PSS, as our previous study demonstrated that no significant changes were found between glycerol concentration and PEDOT:PSS conductivity [22].

### 2.1. Hydrogel Morphology

**Figure 1:**
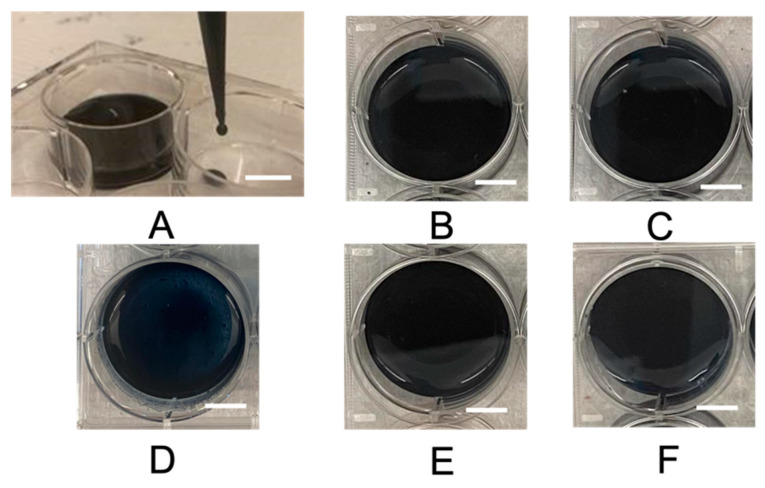
Gross hydrogel morphology after crosslinking. (**A**) An image depicting the water-like consistency of the interpenetrating conductive hydrogel. Gross images of (**B**) NPN, (**C**) LPN, (**D**) NPL, (**E**) NPH, and (**F**) LPH after polymerization. The scalebars represent 1 cm.

**Figure 2:**
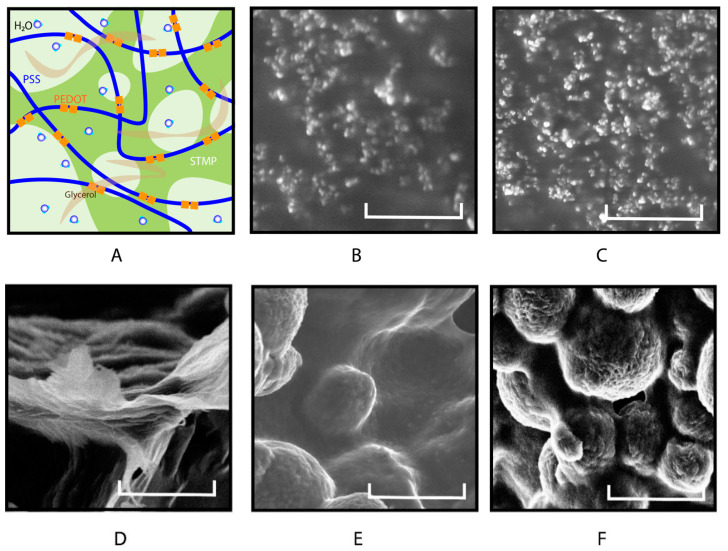
Illustration of the interpenetrating PEDOT:PSS conductive hydrogel structure. (**A**) Schematic illustration of the hydrogel depicting water-dispersed PEDOT:PSS, (1) mixed with glycerol to form strong hydrogen bonds and interfacial crosslinking and (2) with added STMP functioning as a binder that allows free ionic movement. Scanning Electron Microscopy (SEM) image of (**B**) NPN, (**C**) LPN, (**D**) NPL, (**E**) NPH, and (**F**) LPH. The scalebars represent 5 µm.

All the hydrogel components were water-soluble, and no immediate gross changes in the hydrogel solution were observed (Figure 1A). All the conductive hydrogel groups exhibited smooth surface textures after crosslinking (Figure 1). There was no significant variation in the time required for polymerization among all the groups. The physical crosslinking of PEDOT:PSS was based on the excessive negative charge from PSS, which formed ionic bonds with multivalent cations in PEDOT [58]. The rationale for using glycerol is its ability to form strong hydrogen bonds and improve interfacial crosslinking with the PEDOT:PSS complex [59]. The STMP can also serve as an ionic binder that enables the free movement of ions across the hydrogel [50] (Figure 2A). Although chemical crosslinking has many advantages, such as the initiation of an irreversible reaction process that forms covalent bonds resulting in a very stable structure, physical crosslinking minimizes the use of organic reagents that could potentially be toxic for biomedical applications [1]. The physical structure of NPN and LPN revealed that both were thin films with smooth surfaces, as shown in the scanning electron microscopy (SEM) images (Figure 2B,C). NPN and LPN showed uniform coverage of small lumps on the surface under high magnification, consistent with the SEM morphology reported for PEDOT:PSS elsewhere [60]. Our data suggest that glycerol had no impact on the overall physical structure of PEDOT:PSS. In the presence of STMP, it was observed that NPL, NPH, and LPH all exhibited smooth connections between grains on the surface (Figure 2D–F). At low concentrations of STMP, the NPL group demonstrated flocculation with sub-micron thickness (Figure 2D). Without phase separation, the layered composites showed a unique steady combination between STMP and PEDOT:PSS. The NPL composite hydrogels formed a layered structure in comparison to the pristine NPN films with smooth surfaces. STMP crosslinked with PEDOT:PSS to form a scaffold structure [61] at high concentration, as indicated by NPH and LPH (Figure 2E,F). For the LPH group, adding glycerol to STMP-based PEDOT:PSS hydrogels resulted in no significant morphological changes (Figure 2F). The crosslinking between STMP and PEDOT:PSS was successfully confirmed, as evidenced by the high uniformity of grain sizes and porosity observed with SEM (Figure 2E,F) [62]. As suggested by a study that previously reported hyaluronan-STMP-based hydrogel fabrication, the presence of heterogeneous grain sizes with lower porosity indicates insufficient crosslinking [62]. In the synthesis of hyaluronan-STMP-based hydrogels, the stirring speed, high hyaluronan viscosity, and organic phase separation could all prevent and slow down the physical crosslinking reaction [62]. In the present study, we employed all water-soluble components for the synthesis of STMP-based PEDOT:PSS conductive hydrogels, eliminating the aforementioned concerns associated with heterogeneous crosslinking. The presence of STMP changes the physical structure of PEDOT:PSS, which provides the structural foundation for the excellent performance discussed later. These results demonstrate the feasibility of creating interpenetrating PEDOT:PSS conductive hydrogels with glycerol and STMP crosslinking agents. Importantly, small-molecule glycerol has no significant impact on hydrogel morphology, whereas STMP influences the interconnected hydrogel domains at different concentrations.

### 2.2. Conductivity

**Figure 3:**
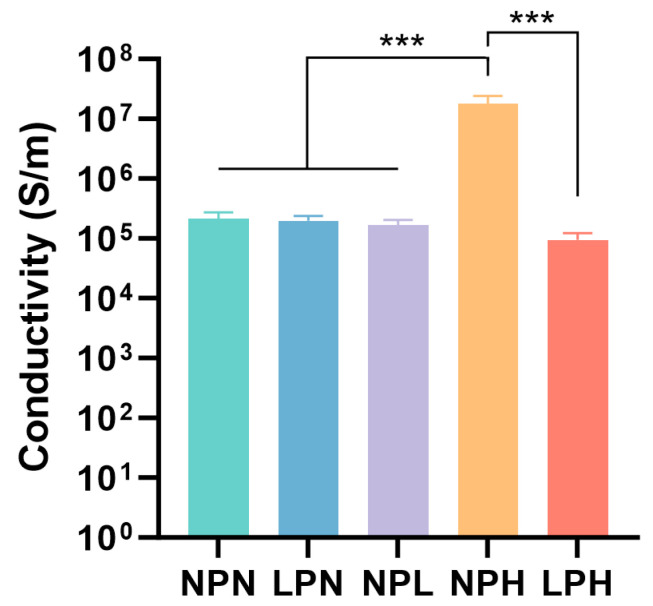
Conductivity measurements of NPN, LPN, NPL, NPH, and LPH hydrogels *** *p* < 0.001.

The PEDOT:PSS polymer has been widely investigated for its biomedical applications due to its favorable electrical conductivity, which improves the adhesion, differentiation, proliferation, and elongation of neural stem cells [8,22,37,63]. We studied the conductivity of our PEDOT:PSS conductive hydrogels composed of different combinations of glycerol and STMP (Table 1). We observed no statistical difference in conductivity between the NPN ((2.13 ± 0.58) × 10^5^ S/m) and LPN ((1.98 ± 0.40) × 10^5^ S/m) groups (Figure 3). This was likely due to the inability of small-molecule glycerol to facilitate significant charge movement at low concentration within the PEDOT:PSS network. The electrical conductivity was mainly contributed to by the charge movement from the crosslinked polymer backbone [64,65,66].

The PEDOT:PSS hydrogels crosslinked with a higher concentration of STMP showed better conductivity (NPH: (1.78 ± 0.63) × 10^7^ S/m) compared to the samples containing low-to-no STMP concentrations (NPL: (1.66 ± 0.37) × 10^5^ S/m) (Figure 3). This was also observed in previous study in which STMP was crosslinked with xanthan gum and silk fibroin to create ion-conductive hydrogels [51]. The crosslinking and hydrogen bonding interactions between xanthan gum, silk fibroin, and STMP enabled the conductive hydrogels to absorb liquid electrolytes and led to surface-dependent ionic conductance [51]. The LPH group, in which both glycerol and STMP were contained in PEDOT:PSS, did not show remarkable changes in conductivity ((9.41 ± 2.83) × 10^4^ S/m) compared to the glycerol-only LPN group ((1.98 ± 0.40) × 10^5^ S/m) (Figure 3). Although STMP concentration could be the standalone factor for increased conductivity, the addition of glycerol was incapable of further enhancing such an effect. It is possible that STMP was stably incorporated into PEDOT:PSS (Figure 2F), exerting long-lasting effects on conductivity [65]. As a commonly used crosslinker, STMP promotes the transformation of the hydroxyl groups of polysaccharides into alcoholate groups in alkaline conditions [67]. It is likely that STMP contributes to increased conductivity by improving the connectivity between the PEDOT:PSS domains [30]. The structure of STMP consists of an isolated (P_3_O_9_)^3−^ cluster network connected through NaO_5_ polyhedrons, with Na^+^ atoms coordinated to oxygen atoms [50]. This distinct inter- and intra-connected polyhedron provides an excellent 3D path for Na^+^ and other ions to diffuse in STMP [50], as well as within the PEDOT:PSS network (Figure 2A). Our data demonstrate that a high concentration of STMP crosslinks the PEDOT:PSS network to increase material conductivity. Because cell culture conditions [68] contain major salts and inorganic ions, such as Na^+^ and Ca^2+^, that can freely enrich and diffuse through the interpenetrating polymer network, we suspect that the conductivity effect of STMP-based PEDOT:PSS hydrogels will be further enhanced when used for cell culture studies. The high liquid and ionic retention induced by STMP will be crucial to ensure the excellent ionic conductivity of hydrogels [69].

### 2.3. Wettability Analysis

**Figure 4:**
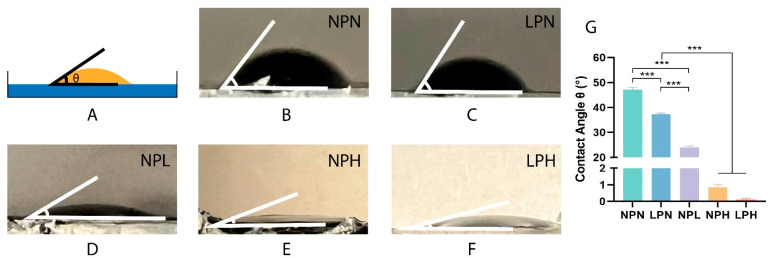
Contact angles for surface wettability. (**A**) Illustration of the wettability analysis. (**B**) NPN, (**C**) LPN, (**D**) NPL, (**E**) NPH, and (**F**) LPH contact angle images. (**G**) NPN, LPN, NPL, NPH, and LPH hydrogels *** *p* < 0.001.

The wettability of different hydrogel formulations was analyzed via contact angle measurements, as illustrated in Figure 4A. All the hydrogel surfaces demonstrated hydrophilic behavior (Figure 4B–F). Introducing a low concentration of glycerol led to decreased contact angles (LPN: 37.23 ± 0.51°) compared to PEDOT:PSS alone (NPN: 47.16 ± 0.80°) (Figure 4G). Similar outcomes were observed when STMP was crosslinked with the PEDOT:PSS polymer network. Increasing the concentration of STMP contributed to smaller contact angles (NPL: 23.96 ± 0.64°, NPH: 0.84 ± 0.16°) (Figure 4G). These data suggest that glycerol and STMP can individually contribute to the hydrophilic surface properties of the conductive hydrogels. The LPH group, in which glycerol and STMP were both added to PEDOT:PSS, demonstrated an extremely small contact angle (0.11 ± 0.07°) (Figure 4G). This illustrates that strong hydrophilicity could be achieved by combining glycerol and a high concentration of STMP. Surface hydrophilicity was positively correlated with an increasing STMP concentration. The wettability of a hydrogel surface is known to influence cell attachment and adhesion, with significant implications for studying cell–material interactions and developing next-generation neural implants [70,71,72]. Previous studies have demonstrated that cell adhesion is more favorable on hydrophilic surfaces [73,74,75]. It was found that osteoblast adhesion decreased significantly when the contact angle increased from 0° to 106° [73]. However, extreme conditions, such as the super-hydrophilic matrix surface (with a contact angle of less than 5°) and super-hydrophobic matrix surface (with a contact angle of more than 150°), are not conducive to cell attachment and growth. Surface wettability influences protein binding (e.g., type, conformation, and strength) from the culture medium, which further affects cell behavior. Hydrophobic surfaces allow protein adsorption in a denaturing manner, which is not suitable for cell binding. Highly hydrophilic surfaces inhibit the binding of cell adhesion mediators, preventing cell attachment. Our data suggest that conductive hydrogels with excellent hydrophilic properties can be obtained when glycerol and STMP are sufficiently crosslinked within the PEDOT:PSS polymer network. It will be important to control the individual crosslinking agents to regulate the hydrogel surface properties for potential biomedical applications.

### 2.4. Rheology Analysis

**Figure 5:**
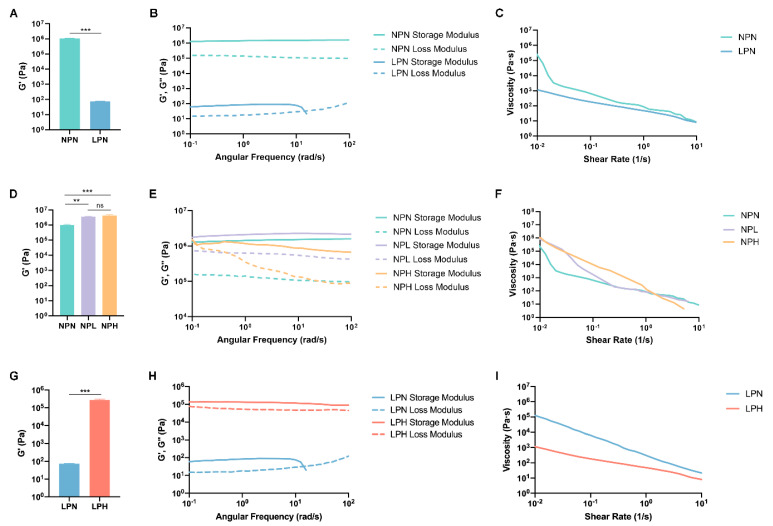
Rheology properties, including stiffness, modulus, and viscosity. (**A**–**C**) NPN and LPN hydrogels. (**D**–**F**) NPN, NPL, and NPH hydrogels. (**G**–**I**) LPN and LPH hydrogels. The average stiffness is the storage modulus within the linear viscoelastic region. All x-y plots are representative samples ** *p* < 0.01, *** *p* < 0.001, ns: not significant.

The rheology of different conductive hydrogel formulations was examined to evaluate the elastic modulus and viscosity properties by conducting logarithmic frequency sweep and flow sweep tests. The stiffness of the LPN hydrogels ((7.33 ± 0.50) × 10^1^ Pa) significantly decreased due to the introduction of glycerol compared to the NPN group ((1.03 ± 0.07) × 10^6^ Pa) (Figure 5A). Both the storage and loss modulus of the LPN hydrogels were much less than those of the NPN group (Figure 5B). In addition, the LPN hydrogels demonstrated lower viscosity, which remained at a lower rate of decline as the shear rate increased (Figure 5C). These results aligned with our study, where added glycerol was used to improve the flexibility of PEDOT:PSS-based soft electronics [22]. Previously, mechanical improvements were noted when low concentrations of glycerol were added to the interpenetrating polymer network to generate wearable electronics that remained relatively stable [41]. It was reported that glycerol interspacing in polysaccharides increases the chain distance, enhancing material elongation, solubility, and smoothness [76]. These findings indicate the importance of using glycerol to increase the overall mechanical flexibility while maintaining the stability of the PEDOT:PSS network.

By crosslinking PEDOT:PSS with different concentrations of STMP, we observed increasing stiffness in both the NPL ((3.54 ± 0.28) × 10^6^ Pa) and NPH ((4.09 ± 0.75) × 10^6^ Pa) groups. Although no significant difference in stiffness was found between the NPL and NPH hydrogels due to concentration changes (Figure 5D,E), the NPH hydrogels demonstrated higher viscosity at lower shear rates (Figure 5F). Our results demonstrate that the presence of STMP significantly increases PEDOT:PSS hydrogel stiffness (NPL and NPH), but changing its concentration can potentially impact the hydrogel mechanical response. Similar effects on STMP concentration were observed in the literature for pullulan–STMP hydrogels [77]. The mechanical behavior of pullulan–STMP hydrogels changes as a result of the amount of STMP crosslinking. In the presence of low-to-no STMP (from 0 to 0.12 molar), the composite hydrogels exhibit increased elastic modulus [77]. However, the elastic modulus plateaus with moderate amounts of STMP (from 0.12 to 0.3 molar). At higher concentrations of STMP (from 0.3 to 0.5 molar), the elastic modulus of pullulan–STMP hydrogels does not continue to change. This is explained by the possible increase in negative charge from phosphorus, which could result in electrostatic repulsions, slowing down the crosslink yield at higher STMP concentrations [77]. The contribution of negative charge functions brought by the STMP could also result in increased swelling, as previously reported [47,51,77]. The LPH group, containing both glycerol and STMP in the PEDOT:PSS hydrogel, demonstrated remarkably higher levels of stiffness ((2.75 ± 0.31) × 10^5^ Pa), as evidenced by its greater storage and loss modulus compared to the LPN hydrogel ((7.33 ± 0.50) × 10^1^ Pa) (Figure 5G,H). Furthermore, lower viscosity and a lower rate of decline in viscosity were found for LPH hydrogels compared to the LPN group (Figure 5I). Our earlier results in this study showed that STMP improved the connectivity between the conductive PEDOT:PSS domains, as evidenced by hydrogel morphology (Figure 2F) and conductivity (Figure 3). The physical crosslinking of STMP within PEDOT:PSS further improved the mechanical properties of the hydrogel by increasing its stiffness and storage modulus [30,78,79,80,81].

The ability to control the rheological properties of conductive hydrogels is important to address the mechanical mismatch that exists between biomedical implants and native biological tissues. Matching the stiffness of implanted biomaterials to that of native tissues can be extremely important for biomaterial-directed cell behavior in tissue engineering and regenerative medicine [22,82,83]. For example, cartilage is a complex nonlinear, viscoelastic, and anisotropic extracellular matrix. STMP has been used to crosslink with polyvinyl alcohol (PVA) to obtain composite hydrogels with mechanical behavior (e.g., G′ changed from 0.01 MPa (0.01 Hz) to 0.02 MPa (15 Hz)) similar to that of tibia cartilage (e.g., G′ = 0.03 and 0.11 for the tissue surface and overall tissue, respectively) [83]. The large mechanical mismatch in stress and Young’s modulus at the interface between the soft nerve and stiff cuff electrode (e.g., silicon rubber, platinum electrode) deteriorates nerve functionality [84,85]. We created glycerol and PEDOT:PSS viscoplastic electrodes that induced zero stress at a lower strain rate of 0.05%/s when applied to the normal growing sciatic nerve (e.g., 2 × 10^−5^%/s) [22]. Our data suggest that STMP increases the elastic performance, whereas glycerol improves the viscous properties of the PEDOT:PSS-based conductive hydrogel.

The increasing viscous properties due to glycerol could be effectively reversed by inducing a higher concentration of STMP. This reversal may be important in the 3D bioprinting of conductive-based hydrogels for potential biomedical applications. In particular, the shear-thinning behavior demonstrated by our STMP-based conductive hydrogels (Figure 5) could be useful for extrusion-based 3D bioprinting [86]. Highly viscous hydrogels encapsulating living cells experience higher shear stress deposition through the printing nozzle, which negatively impacts the viability of the encased cells. Therefore, developing hydrogels that exhibit shear-thinning behavior is essential for the extrusion-based 3D bioprinting process to support encapsulated cells and maintain printed geometric fidelity [87,88].

## 3. Conclusions

In summary, our findings suggest that the properties of PEDOT:PSS can be controlled by individually adjusting glycerol and STMP additives. Importantly, the conductivity, wettability, and rheological properties of PEDOT:PSS-based hydrogels can be significantly enhanced with the addition of STMP. The increased conductivity and storage modulus produced by STMP physically crosslinking with PEDOT:PSS is more significant than that achieved with small-molecule glycerol (Table 1). The mechanism by which STMP forms a smooth and highly crosslinked structure within the PEDOT:PSS interpenetrating network is the key to improving the conductivity and mechanical strength of STMP-based PEDOT:PSS conductive hydrogels. Furthermore, the shear-thinning rheological behavior demonstrated by the STMP-based PEDOT:PSS conductive hydrogels is typically required for extrusion-based 3D bioprinting to support the viability of the encased cells and geometric structures. Conductive hydrogels provide a platform to control cell functions through electrical, chemical, and physical cues presented in the cellular microenvironment [6]. Conductive hydrogels with tunable material properties are highly attractive to researchers due to their ability to recreate physiological conditions that mimic biological processes, model disease progression, and develop new regenerative strategies for tissue and organ repair [21]. Our highly interpenetrating STMP-based PEDOT:PSS conductive hydrogels, with tunable abilities for electrical and mechanical characteristics, can provide physiological environmental cues for future tissue engineering applications.

## 4. Materials and Methods

### 4.1. Materials

Glycerol with a purity of over 99.5% (catalog: G9012) and a surfactant-free poly(3,4-ethylenedioxythiophene)-poly(styrenesulfonate) (PEDOT:PSS) aqueous dispersion with 1.1 wt% in H_2_O (catalog: 739332) were purchased from Sigma-Aldrich. Sodium trimetaphosphate (STMP) (catalog: AA89063A1) was purchased from Thermo Fisher Scientific. Deionized (DI) water (>18.2 MΩ) was used throughout this study.

### 4.2. Synthesis of Conductive Hydrogel Formulations

The synthesis procedure for glycerol-added PEDOT:PSS hydrogels was modified from our previous publication [22]. Briefly, a series of solutions were formulated at room temperature with varying volume ratios of glycerol and STMP in a constant volume ratio of PEDOT:PSS for all experimental formulations (Table 1). PEDOT:PSS remained at a constant volume ratio (8 µL) for all the groups. The glycerol formulations were prepared with 1 wt% glycerol in a volume ratio of either no glycerol (0 µL) or with a low concentration (1 µL). STMP was dissolved in DI water to obtain a 22 wt% solution and was utilized in either no STMP (0 µL), low concentration (1 µL), or high (15 µL) concentration volume ratios. There were a total of five hydrogel formulations prepared with the following ratios of glycerol, PEDOT:PSS, and STMP: 0:8:0 (NPN), 1:8:0 (LPN), 0:8:1 (NPL), 0:8:15 (NPH), and 1:8:15 (LPH) (Table 1). Each hydrogel solution was covered with aluminum foil and stirred vigorously for 25 min at a speed of 120 rpm until homogenized. All the experimental groups formed solid-like hydrogels upon resting at room temperature.

### 4.3. Morphological Analysis

To perform the scanning electron microscopy (SEM) image analysis, all the samples were first frozen in −80 °C overnight and then lyophilized for 24 h. The samples were then cut into small pieces and transferred onto sample holders for SEM imaging. The imaging process was mainly based on the detection of secondary electrons emerging from the surface under the impact of a very fine beam of primary electrons scanning the observed surface. The Hitachi S-4800, equipped with a cold-field emission gun (cold FEG) operating between 5 eV to 20 eV, was used throughout this study. We were interested in using this type of microscope because of its large range of magnification, spanning from ×20 to ×800 K. The combined use of the super ExB filter with the first upper detector allowed the filtering and collection of secondary electrons and backscattering signal energies of interest, thereby suppressing charging artifacts and revealing topographical details. The resolution was limited to 1 nm at 15 kV and 1.4 nm at 1 kV.

### 4.4. Conductivity Measurement

For conductivity testing, 2 mL of each sample solution was directly added to the wells of a tissue culture plate (Genesee Scientific, EI Cajon, CA, USA). The plate was covered with aluminum foil and left at room temperature to solidify. After polymerization, the samples were measured using a digital four-point probe tester (JG Suzhou Jingge Electronic Co., Ltd., Suzhou, China) with corresponding correction coefficient values based on the manufacturer’s software (v1.2). Specifically, the diameter (mm) and sample thickness (mm) of each circular sample were measured using a caliper and were entered into the four-point probe. The four-point probe was gently lowered into the hydrogel and the resistance was recorded once every ten seconds for a total of ten points per individual well sample. All the samples were measured at room temperature. Conductivity was calculated as the inverse of sample resistivity.

### 4.5. Hydrophilicity Analysis

After the samples were polymerized to form solid hydrogels, we deposited a single droplet of DI water onto the hydrogel surfaces. At least three pictures were captured in the horizontal position with a camera. ImageJ software (v1.61) was used to determine the angle of the water droplet with the hydrogel surface. The contact angle indicates the wettability of the sample surface.

### 4.6. Rheological Measurement

A customized 8-mm diameter well plate was filled with each hydrogel solution. The well plates were covered with aluminum foil and left at room temperature until solidified. The hydrogels were tested using the Discovery Hybrid Rheometer 2 (TA Instruments, New Castle, DE, USA) with a humidity chamber to prevent dehydration over the course of the experiment. The results were analyzed using TRIOS software (v5.2). To demonstrate hydrogel formation, a time-sweep was performed at 23 °C with an oscillatory strain of 1% at an angular frequency of 1 rad/s over the course of 30 min. Logarithmic frequency sweep tests were performed at a strain of 1% within the linear viscoelastic region. The storage modulus (G′) and loss modulus (G″) were recorded as a function of angular frequency. Logarithmic flow sweep tests were performed to record the apparent viscosity as a function of shear rate. All the measurements were performed at room temperature using an 8-mm parallel-plate steel geometry. The average storage modulus and stiffness were determined from the linear viscoelastic region of the oscillation frequency sweep curves. In the present experiments, at least three independent samples were measured and the values were averaged to represent the matrix stiffness.

### 4.7. Statistical Analysis

At least three samples were applied for the experiments in each group. An independent Student’s *t*-test was used for the comparison between two groups, one-way ANOVA was used to compare the differences among multiple groups, and Tukey’s multiple comparisons test was employed to evaluate between-group differences. The data analysis was performed using GraphPad Prism 10 (v10.1.1), and a *p* value < 0.05 was considered statistically significant in this study. The data were presented as mean ± standard error of the mean.

## Figures and Tables

**Table 1:** Summary of sample constituents and measurements.

	Glycerol (vol. Ratio)	PEDOT:PSS (vol. Ratio)	STMP (vol. Ratio)	Conductivity (S/m)	θ (°)	Stiffness (Pa)
NPN (Control)	0	8	0	(2.13 ± 0.58) × 10^5^	47.16 ± 0.80	(1.03 ± 0.07) × 10^6^
LPN	1	8	0	(1.98 ± 0.40) × 10^5^	37.23 ± 0.51	(7.33 ± 0.50) × 10^1^
NPL	0	8	1	(1.66 ± 0.37) × 10^5^	23.96 ± 0.64	(3.54 ± 0.28) × 10^6^
NPH	0	8	15	(1.78 ± 0.63) × 10^7^	0.84 ± 0.16	(4.09 ± 0.75) × 10^6^
LPH	1	8	15	(9.41 ± 2.83) × 10^4^	0.11 ± 0.07	(2.75 ± 0.31) × 10^5^

## Data Availability

The data presented in this study are openly available in this article.
